# Localized Therapy of Vaginal Infections and Inflammation: Liposomes-In-Hydrogel Delivery System for Polyphenols

**DOI:** 10.3390/pharmaceutics11020053

**Published:** 2019-01-27

**Authors:** May Wenche Jøraholmen, Purusotam Basnet, Mia Jonine Tostrup, Sabrin Moueffaq, Nataša Škalko-Basnet

**Affiliations:** 1 Drug Transport and Delivery Research Group, Department of Pharmacy, Faculty of Health Sciences, University of Tromsø The Arctic University of Norway, Universitetsveien 57, 9037 Tromsø, Norway; may.w.joraholmen@uit.no (M.W.J.); miatostrup@icloud.com (M.J.T.); sabrin.moueffaq@hotmail.com (S.M.); 2 IVF Clinic, Department of Obstetrics and Gynecology, University Hospital of North Norway, Sykehusvegen 38, 9019 Tromsø, Norway; purusotam.basnet@uit.no; 3 Women’s Health and Perinatology Research Group, Department of Clinical Medicine, University of Tromsø The Arctic University of Norway, Universitetsveien 57, 9037 Tromsø, Norway

**Keywords:** polyphenols, resveratrol, epicatechin, liposomes, hydrogel, chitosan, vaginal drug delivery

## Abstract

Natural polyphenols, such as resveratrol (RES) or epicatechin (EPI), are attractive for treatments of various diseases, including vaginal infections and inflammation, because of their strong anti-oxidative and anti-inflammatory properties. However, their low solubility and consequent poor bioavailability limit their therapeutic uses. To overcome these limitations, a vaginal delivery system comprising either RES or EPI liposomes-in-hydrogel was developed. This system permits therapeutic action of both liposomal polyphenol (RES or EPI) and chitosan-based hydrogel. Liposomes of around 200 nm and entrapment efficiency of 81% and 77% for RES and EPI, respectively, were incorporated into chitosan hydrogel, respectively. Medium molecular weight chitosan (2.5%, *w*/*w*) was found to have optimal texture properties and mucoadhesiveness in ex vivo conditions. The in vitro release studies confirmed the sustained release of polyphenols from the system. Both liposomal polyphenols and polyphenols-in-liposomes-in-hydrogel exhibited only minor effects on cell toxicity. EPI showed superior radical scavenging activity at lower concentrations compared to antioxidants vitamin C and E. Anti-inflammatory activity expressed as the inhibitory activity of formulations on the NO production in the LPS-induced macrophages (RAW 264.7) confirmed the superiority of EPI liposomes-in-hydrogel. The plain liposomes-in-hydrogel also exhibited potent anti-inflammatory activity, suggesting that chitosan hydrogel acts in synergy regarding anti-inflammatory effect of formulation.

## 1. Introduction

Sexually transmitted infections (STIs) are a heterogeneous group of infections affecting millions of people. STIs are among the most common acute conditions worldwide. WHO estimates that one million cases of STIs, namely chlamydia, gonorrhea, syphilis, and trichomoniasis, all curable infections, occur daily [1]. Chlamydia infections and bacterial vaginosis are considered the most common infections of vaginal tract. Failure to treat or cure STIs leads to severe consequences such as infertility, miscarriage, stillbirth, and transmission of infection from mother to child. Yet, those infections are rather a neglected health topic and current therapies fail to manage the treatment effectively. To add to the already limited treatment success is the emerging spread of antimicrobial resistance, especially in *Neisseria gonorrhoeae* [2]. 

In past years, we have been focused on two approaches to address the failure of management of STIs, namely the development of advanced delivery systems for optimized localized vaginal therapy and use of natural origin substances as antimicrobial and anti-inflammatory agents [3,4,5]. 

Polyphenols are gaining increased attention as substances of natural origin with multifunctional properties and have been extensively studied as potential treatments of various diseases. Resveratrol (RES) is one of the polyphenols of natural origin, mainly from blueberry or red grapes, with multifunctional therapeutic effects. It is a member of stilbene family, widely studied for its biological properties, mostly attributed to its anti-oxidative, anti-inflammatory, and immune-modulating effects [6]. Recently, its effects on uterine scarring and the remodeling of scarred uterus have been proven. RES not only decreased inflammation, but also improved the pregnancy outcome [7]. However, RES suffers from rapid metabolism and low bioavailability when given orally, therefore, various nano-formulations have been proposed as means to overcome RES limitations as therapeutic agents [6]. Interestingly, very recent publication by Tang and colleagues claims that RES acts as an effective alpha-hemolysin (Hla) inhibitor that reduces Hla expression without antimicrobial activity and could serve as a potent agent against *Staphylococcus aureus* infections [8]. On the other hand, most of the polyphenols present in green tea are flavanols, commonly known as catechins. Tea catechins have many health benefits, such as anti-inflammatory, antiarthritic, anticarcinogenic, antimutagenic, antibacterial, antiviral, antifungal, anticoccidial, antiprotozoal, antiparasitic, anti-infective, hypocholesterolemic, and hypolipidemic effects [9]. In 2006, the green tea extract was approved by the Food and Drug Administration (FDA, USA) for the treatment of genital warts. It is the first botanical extract ever approved by the FDA as a prescription drug, shown to be superior in clinical studies, compared to conventional genital warts therapy [10]. Epicatechin (EPI) is one of the several recently proposed anti-inflammatory interventions beneficial in alleviating the rapid progression of tissue damage. Although its anti-oxidative properties are widely recognized, its anti-inflammatory effects remain to be explored, especially at the mechanistic level. Recently, EPI was proven to alleviate inflammatory lung injury by inhibiting the p38 MAPK signaling pathway [11]. To overcome poor solubility and often instability of polyphenols, various approaches, including nanotechnology, have been proposed. Considering the conditions the formulation is facing once administered vaginally [12], the choice of a suitable carrier needs to be carefully evaluated, both regarding the safety and potential irritancy of the formulation and ability to retain active ingredient at the vaginal site at sufficiently high concentrations over a longer time.

**Figure 1:**
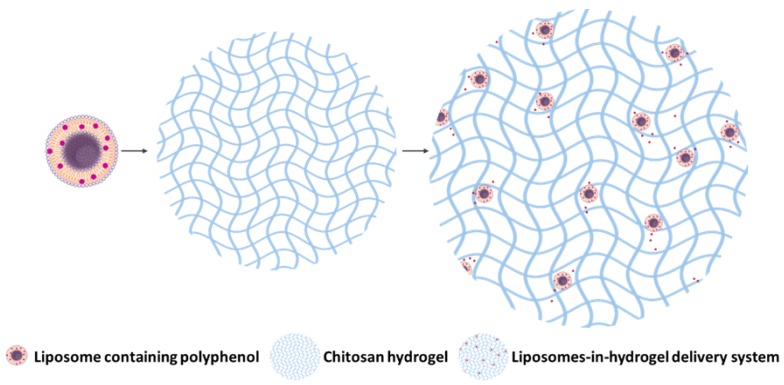
Graphical description of the liposomes-in-hydrogel delivery system for model polyphenol.

Liposomes have been shown as suitable nanocarriers for polyphenols [3,5]. However, as a colloidal dispersion in aqueous medium they require a suitable semisolid vehicle to assure optimal applicability, spreadability, and retention time at the vaginal site [13]. Existing semi-solid formulations, such as creams and hydrogels, for localized vaginal therapy, are mostly based on synthetic polymers [14]. These formulations are rather easy to administer, mostly accepted by patients/users, and relatively affordable. However, they are also rather messy and suffer from pre/post leakage issues [15]. Due to the self-cleansing action of the vaginal tract, many semi-solid formulations fail to assure sustained drug release at the vaginal site. The semi-solid formulations therefore require at least once daily dosing to assure a therapeutic outcome [16]. Hydrogels are mostly prepared as vehicles for hydrophilic drugs (active ingredients) [17], however, by incorporating nanosystems as carriers for either/both lipophilic and hydrophilic substances, their attractiveness is increasing. Moreover, by using natural-origin polymers such as chitosan, their biocompatibility and safety is improving; chitosan is especially attractive due to its intrinsic biological properties. Furthermore, chitosan can provide a controlled release of incorporated active ingredient, and combined with its mucoadhesive properties and intrinsic antimicrobial activity, chitosan appears as an excellent excipient in vaginal drug delivery [18,19]. The combination of two delivery systems (Figure 1), one enabling the incorporation of poorly soluble substances (liposomes) and another assuring the prolonged contact time at the site of drug action (hydrogels), represents a novel and promising approach in optimization of localized drug therapy, particularly suitable for vaginal administration. If, as in our case, the secondary system, hydrogel, possesses intrinsic antimicrobial and potentially anti-inflammatory properties, this would greatly improve the therapeutic outcome.

In the present work, we focused on development of chitosan-based liposomes-in-hydrogel delivery system for model polyphenols, RES and EPI. Each of the polyphenol, having similar biological effects while of different chemical structures, was singularly incorporated in novel formulation and their properties were compared. We have earlier reported that incorporation of RES in liposomes enhances its biological activity [5]. We have now moved step further to develop a final formulation, namely either RES liposomes-in-hydrogel or EPI liposomes-in-hydrogel. The novel hydrogels were fully characterized, compared and their potential for vaginal therapy evaluated. 

## 2. Materials and Methods 

### 2.1. Materials

Lipoid S 100 (>94% phosphatidylcholine) was a gift from Lipoid GmbH, Ludwigshafen, Germany. Chitosan (medium molecular weight hydramer HCMF) was a gift from Chitinor, Tromsø, Norway. Resveratrol (RES: 3,5,4′-trihydroxy-trans-stilbene, purity ≥99%), epicatechin (EPI: (−)-epicatechin, purity ≥90%), 2,2′-azino bis(3-ethylbenzothiazoline)-6-sulfonic acid diammonium salt (ABTS), 1,1-diphenyl-2-picrylhydrazyl (DPPH), vitamin C (ascorbic acid), vitamin E, MTT (3-(4,5-dimethylthiazol-2-yl)-2,5-diphenyl-2H-tetrazolium bromide), RPMI medium 1640, acetic acid, glutamine, glycerol, sodium chloride, sodium dihydrogen phosphate, propylene glycol, bovine serum albumin, calcium hydroxide, and glucose were purchased from Sigma-Aldrich, Chemie GmbH, Steinheim, Germany. Lipopolysaccharide (LPS; *Escherichia coli*, 055:B5) was purchased from Sigma Life Science (Sigma–Aldrich Norway AS, Oslo, Norway). Potassium peroxodisulphate was a product from Merck KGaA, Darmstadt, Germany. Ammonium acetate was purchased from VWR International, Leuven, Belgium. Potassium hydroxide, lactic acid and urea were from NMD, Oslo, Norway. Griess reagent was prepared from 1% sulfanilamide, 0.1% naphthylethylenediamine dihydrochloride, 2.5% phosphoric acid; all products were from Sigma-Aldrich Norway AS, Oslo. HaCaT cells were supplied from Thermo Fisher Scientific, Waltham, MA, USA and murine macrophages RAW264.7 cells were from ATCC, Manassas, VA, USA.

### 2.2. Preparation of Liposomes 

Liposomes were prepared by the film hydration method as previously described [5]. Briefly, RES (10 mg) and lipid (Lipoid S 100; 200 mg) were dissolved in methanol. Solvent was removed by evaporation (Büchi rotavapor R-124 with vacuum controller B-721, Büchi Vac^®^ V-500, Büchi Labortechnik, Flawil, Switzerland) for at least 3 h at 50 mm Hg and 50 °C. The remaining lipid film was rehydrated in distilled water (10 mL) and liposomal suspension stored in refrigerator (4–8 °C) overnight prior to further use. EPI liposomes were prepared in a similar manner. Plain liposomes were prepared using only Lipoid S 100 (200 mg).

Liposomal size was reduced by extrusion through polycarbonate membranes (Nuclepore Track-Etch Membrane, Whatman House, Maidstone, UK). The extrusion was done stepwise through 0.8, 0.4 and 0.2 µm and 0.1 µm pore size filters and repeated five times for each step [5]. Liposomes were then stored in a refrigerator for at least 6 h prior to further use.

### 2.3. Characterization of Liposomes

#### 2.3.1. Vesicle Size

Photon correlation spectroscopy (Submicron particle sizer model 370, Nicomp, Santa Barbara, CA, USA) was used to determine the vesicle size and polydispersity of liposomal suspensions. The work was performed in a laminar airflow bench as described earlier [20], and analyses were run in the vesicle mode and intensity-weight distribution. Each sample was measured in triplicate of a run time of 10 min.

#### 2.3.2. Zeta Potential

Zeta potential was determined by Malvern Zetasizer Nano Z (Malvern, Oxford, UK). Measurement cells (DTS1060) were rinsed with ethanol and filtrated water (0.2 µm) prior to loading of sample. Samples were prepared and measured in triplicates at 25 °C as previously described [21]. 

#### 2.3.3. Polyphenol Entrapment Efficiency

Free polyphenol was separated from liposomally entrapped polyphenol by dialysis using dialysis tubing (Mw cutoff: 12–14,000 Da, Medicell International Ltd., London, UK). A ratio of 1:250 mL (liposomal sample:dialysis medium, distilled water) was used [5]. Aliquots of both sample and medium were diluted in methanol, transferred to a microtitre plate (Costar^®^UV 96-well plate with a UV-transparent flat bottom, Acrylic, Costar^®^, Corning, New York, NY, USA) and RES and EPI, determined spectrophotometrically (Microtitre plate reader; Spectra Max 190 Microplate, Spectrophotometer Molecular devices, Sunnyvale, CA, USA) at 306 and 280 nm, respectively. 

### 2.4. Preparation of Hydrogels

#### 2.4.1. Chitosan Hydrogels 

Chitosan hydrogels were prepared according to previously described method [22] with slight modifications to adapt the gel to the vaginal environment. In brief, appropriate amounts of medium molecular weight chitosan were dissolved in the mixture containing 2.5% acetic acid and 10% (*w*/*w*) glycerol, followed by 48 h swelling at room temperature (23–25 °C).

#### 2.4.2. Preparation of Liposomes-In-Hydrogel Formulation

Liposomes containing polyphenols (RES liposomes or EPI liposomes, free from unentrapped RES and EPI) were carefully mixed into chitosan hydrogels by a hand stirring until evenly distributed in the hydrogel. Formulations with a final concentration of 10% and 20% (*w*/*w*) liposomal suspension were prepared.

### 2.5. Preparation of Vaginal Tissue

For mucoadhesion testing, the cow vaginal tissue obtained from the local slaughter house (Nortura, Målselv, Norway) was used. The vaginal tissue from pregnant sheep obtained from the Laboratory Animal Centre, University of Oulu, Finland was used in the ex vivo penetration studies (National Animal Experiment Board in Finland ESAVI/3510/04.10.03/2011). The vaginal tissue was carefully separated from underlying tissue, rinsed and moistened with phosphate buffer (PBS, pH 7.4, 8.0 g/L NaCl, 0.19 g/L KH_2_PO_4_, and 2.38 g/L Na_2_HPO_4_), packed in clinging film and frozen (–20 °C). Prior to experiments, the tissue was left to defrost in PBS for up to 1 h. 

### 2.6. Characterization of Hydrogels

#### 2.6.1. Texture Analysis

The texture properties (cohesiveness, adhesiveness and hardness) of the hydrogels were determined by the backwards extrusion using Texture Analyzer TA.XT plus (Stable micro systems Ltd., Surrey, UK). The method established in our laboratory [23] was applied with minor modifications. Briefly, a probe disk (40 mm) was compressed into the hydrogel (40 g) at a speed of 4 mm/s for a distance of 10 mm. Five measurements were taken for each sample.

#### 2.6.2. Mucoadhesive Properties

A mucoadhesion rig for the Texture Analyzer TA.XT plus (Stable micro systems Ltd., Surrey, UK) was used in the determination of the ex vivo mucoadhesive properties of the hydrogels [22]. Cow vaginal tissue was cut to appropriate sized pieces, rinsed with ethanol and phosphate buffer, respectively, and clamped onto the membrane holder. Hydrogel (150 µL) was applied to the probe associated to the mucoadhesion rig. The probe was compressed onto the mucosal tissue with a force of 25 g for 10 s and redrawn with a speed of 0.1 mm/s and the detachment force was measured. Further, the amount of formulation retained on the tissue was determined by weighing the probe prior to and after a compression. The vaginal tissue was rinsed with ethanol and PBS between every measurement and 5 parallels determined for all samples.

#### 2.6.3. In Vitro Polyphenol Release

The in vitro polyphenol release was determined according to the method described previously [20] using the Franz cell manual diffusion system (Perme Gear Ink, Diffusion cells and Systems, Hellertown, PA, USA). The receptor chamber (12 mL) was filled with acetate buffer (pH 4.6, 77.1 g/L CH_3_COONH_4_, 70 mL glacial acetic acid) and the heating circulator (Julabo Laboratechnik, F12-ED, Seelback, Germany) was set to 37 °C. Cellophane pre-soaked in acetate buffer served as a membrane. Formulations and controls (600 µL), all containing similar RES or EPI concentrations, respectively, were evenly distributed in the donor chambers. Samples (500 µL) were withdrawn from the receptor chambers after 1, 2, 3, 4, 6, and 8 h and replaced by an equal volume of fresh buffer. Experiments were performed in triplicates and the amount of RES and EPI was determined spectrophotometrically at 306 nm and 280 nm, respectively.

#### 2.6.4. Preparation of Sheep Vaginal Tissue

Prior to the experiments, the tissue was left to defrost in phosphate buffer (pH 7.4) at room temperature for at least one hour. Tissue was cut to appropriate size and the thickness was measured to confirm that it was in the range of 900–1100 µm. The tissue was applied in penetration studies.

#### 2.6.5. Ex Vivo Penetration

The experiment was performed on the Franz cell manual diffusion system as described above, with phosphate buffer as receptor medium and sheep vaginal tissue as a barrier [21]. Formulations and controls (550 µL) and vaginal fluid simulant (VFS, 50 µL, pH 4.6, 3.51 g/L NaCl, 1.40 g/L KOH, 0.222 g/L Ca(OH)_2_, 0.018 g/L bovine serum albumin; 2 g/L lactic acid, 1 g/L acetic acid, 0.16 g/L glycerol, 0.4 g/L urea, and 5 g/L glucose [24]) were added in the donor chambers. The aliquots (500 µL) were withdrawn from the receptor chambers after 1, 2, 3, 4, 6, and 8 h and replaced by an equal volume of fresh buffer. RES and EPI content in all samples was determined by HPLC (methods described below). Experiments were performed in triplicate. 

#### 2.6.6. Polyphenol Quantification by HPLC

RES content was determined by the HPLC method. A reversed phase column (Symmetry^®^ C18 5 µm 3.9 × 150 mm column, Waters, Dublin, Ireland) installed in a Waters e2795 separations module coupled with a Waters 2489 UV–VIS detector were used. The mobile phase consisted of 75% MeOH, 22.5% acetonitrile, 2.4% MilliQ water, and 0.1% of acetic acid [25]. Amount of RES was detected at 306 nm with a flow rate of 0.8 mL/min, column and sample temperature of 25 °C, an injection volume of 20 µL and a run time of 5 min. 

For determination of EPI content, a similar HPLC method was used as described above, except the mobile phase comprised 0.1% TFA in MilliQ water (pH 2.0) mixed with methanol in a ratio 75:25 (*v*/*v*); a run time of 13 min was used and detection was performed at 280 nm [26]. 

All measurements were performed in triplicates.

### 2.7. Cell Toxicity

The in vitro toxicity was evaluated on keratinocytes (HaCaT cells) by measuring cell viability after treatment by the liposomal hydrogel formulations. The cells were cultured in RPMI medium supplemented with glutamine and 10% serum at humidified 5% CO_2_ and 37 °C. The cytotoxicity was tested by MTT assay method with cell proliferation kits following the instruction provided by the supplier. In brief, 90 µL of trypsinized HaCaT cell suspension (5 × 10^5^ cells/mL) were plated in the flat bottom 96 wells plate. To each well 10 µL media only (control) or the samples diluted in media (resulting in a final concentration of 1, 10, or 50 µg/mL, respectively) were added. The cells were incubated for 24 h. The next day, 10 µL of MTT (with a final concentration of 0.5 mg/mL) was added to each well and the cells incubated for 4 h for the development of violet crystals of formazan. After 4 h, 100 µL of solubilizing reagents (supplied in the assay kits) was added to each well and the cells were kept in the incubator for 24 h. The UV absorption of soluble formazan was determined on ELISA plate reader at 580 nm. The UV absorption determined for the control group was considered as 100% viable and the effects of various concentration of tested samples on cell toxicity were expressed as percentage viability as compared to the control. Results are expressed as the mean of two independent experiments done in triplicates for each sample.

### 2.8. Anti-Oxidative Activities

#### 2.8.1. DPPH Radical Scavenging

Anti-oxidative effect of EPI was evaluated by DPPH free radical scavenging activity as described previously [3]. Equal volumes (0.3 mL) of DPPH solution (60 µM) and EPI solutions (5, 10, 25, 50, and 75 µM as final concentrations) were mixed thoroughly and kept in dark for 30 min at room temperature. As DPPH radical concentrations decrease due to EPI anti-oxidative reaction, the violet color disappears. The decrease in absorbance intensity correlates to a radical scavenging activity of EPI and was determined spectrophotometrically at 519 nm [5]. The activity was compared under the same conditions to those of vitamins C and E in corresponding concentrations.

#### 2.8.2. ABTS**·**+ Radical Scavenging

Equal volumes (3 mL) of the stock solutions of ABTS (7.4 µM) and potassium peroxodisulphate (2.6 µM) were mixed and left overnight to generate and stabilize ABTS**·**+ radical at room temperature and then diluted in ethanol (100 mL). ABTS**·**+ radical solution (0.3 mL) was mixed thoroughly with equal volumes of EPI solutions (5, 10, 25, 50, and 75 µM as final concentrations) and kept in dark for 30 min at room temperature. The green color of ABTS**·**+ radical disappears as it reacts with antioxidants. Therefore, the decrease of free radical concentration directly expresses the anti-oxidative activity as measured spectrophotometrically at 731 nm. The anti-oxidative activity of EPI was expressed as described for DPPH assay above and also compared to the activity of vitamins C and E.

### 2.9. Anti-Inflammatory Activity Measurement

The in vitro anti-inflammatory activities of formulations were studied by assessing the effects of EPI or RES liposomes-in-hydrogel formulations on the inhibition of NO production in LPS-induced macrophages (RAW 264.7 cells) by similar methods as reported earlier [3]. In brief, macrophages (1 × 10^5^ cells/mL) were incubated in a 24-wells plate with RPMI 1640 medium supplemented containing 10% serum and glutamine for at 37 °C/5% CO_2_. After 24 h, old medium was replaced with new media containing 1 µg/mL LPS to induce inflammation. The formulations at various lipid concentrations, namely 1, 10 and 50 µg/mL, or 1 µg/mL LPS-containing media only (control) were added each with 10 µL volume. After 24 h incubation, NO production by the cells in the media was expressed by measuring the nitrite concentration with Griess reagent by measuring the absorbance at 540 nm using UV spectrophotometer (Agilent Technologies, Santa Clara, CA, USA). The effect of the formulations on inhibition of NO production was expressed as percentage inhibition of produced NO, in comparison to 100% NO detected in the control (cells treated with 1 µg/mL LPS).

### 2.10. Statistical Analyses

Results are expressed as mean and SD, where *n* = 3. For the comparison of two means, statistical significance was determined using the Student’s *t*-test. A *p*-value less than 0.05 was considered statistically significant.

## 3. Results and Discussion

Successful local treatment of vaginal infections depends on several factors besides the drug therapeutic potential, such as a uniform distribution throughout the vaginal cavity and a sufficient drug concentration at vaginal site for an adequate period of time [12]. To fulfill these requirements, the texture properties and mucoadhesiveness of the formulation, as well as a controlled and prolonged release of entrapped active substance, are the prerequisites.

### 3.1. Liposomal Characteristics 

Liposomes as drug delivery system offer numerous advantageous in localized treatment and have been confirmed as suitable carriers for topical skin therapy [27]. Due to their ability to provide sustained and controlled release of entrapped substances, and encapsulate both lipophilic and hydrophilic molecules, liposomes have been proposed as an advanced drug delivery system for vaginal therapy [28]. By tailoring their size, polydispersity and surface characteristics, it is possible to optimize their efficacy as delivery system. In this study, liposomes were characterized for size, size distribution, zeta potential and polyphenol entrapment efficiency (Table 1). 

Liposomal size and size distribution are important characteristics when aiming for localized therapy. It is suggested that nanocarriers in the size range of 200–500 nm are superior to both smaller and lager carriers considering drug delivery to mucosal tissues [29]. We aimed at the vesicles around 200 nm to assure both a depot effect at the vaginal site, carrying a sufficient load of polyphenols, as well as being stable and avoiding potential precipitation during the cell studies. Furthermore, the very low polydispersity index (PI) indicated a rather homogenous size distribution, additionally confirming that the extrusion is a highly suitable size reduction method for liposomal suspensions. The extrusion technique has shown to be effective and reproducible by both manual and mechanical extruders [30]. We previously confirmed that liposome size reduction by the extrusion method did not result in loss of lipids or polyphenols under the applied extrusion protocol [5]. Phosphatidylcholine is a zwitter ionic lipid, exhibiting a negatively-charged phosphate group and a positively-charged choline, resulting in a zeta potential values close to neutral, hence, liposomes made of phosphatidylcholine are expected to exhibit a neutral zeta potential [31]. Both liposomes containing RES and liposomes containing EPI expressed a close to neutral zeta potential (Table 1). This is in agreement with earlier reports [5,32]. A liposomal zeta potential close to neutral would be favorable considering the release of liposomally-associated molecules when liposomes are incorporated in chitosan hydrogel, as well as the textural properties of liposomes-in-hydrogel system [33].

Several groups reported on the improved solubility of RES and EPI by their incorporation into liposomes, therefore increasing their entrapment efficiency and bioavailability [34,35,36]. Both polyphenols expressed a relatively high entrapment efficiency, with an average liposomal entrapment of 81% and 77% for RES and EPI, respectively (Table 1). This corresponds to literature reporting polyphenol entrapment over 70% for both RES [5,25,37] and EPI [38]. In addition to the difference in chemical structures, the liposomal polyphenol entrapment efficiency has shown to vary depending on the production method and lipid composition [36]. In the present work, we used phosphatidylcholine (PC) to assure neutral liposomes and found that the entrapment efficiencies were high and reproducible for both polyphenols (Table 1).

Liposomes as carriers can sustain the stability of entrapped substance by protection from the surrounding environment, thus, also enhance its effectiveness [5]. An increase in the chemical stability of the polyphenols can be achieved, leading to prolonged efficacy [36].

### 3.2. Liposomes-In-Hydrogel Characteristics

The combination of delivery systems opens opportunities for innovative therapeutic strategies. Various combinations of delivery systems can be utilized, targeting various routes of administration and diseases [39]. Although superior in many ways as nanocarriers, liposomes require secondary vehicle to assure their prolonged residence time at vaginal cavity [13]. We have opted for chitosan, a polymer of natural origin, with known mucoadhesive properties [22]. 

#### 3.2.1. Texture and Mucoadhesiveness

Texture analysis provides information on hydrogel properties in a reproducible and validated way. The experimental setup will affect the measurements; hence, it is crucial to establish a reproducible method suitable for the particular type of hydrogel. An improved and simplified method, based on the original method developed in our group, applies the texture analyzer [23]. 

**Figure 2:**
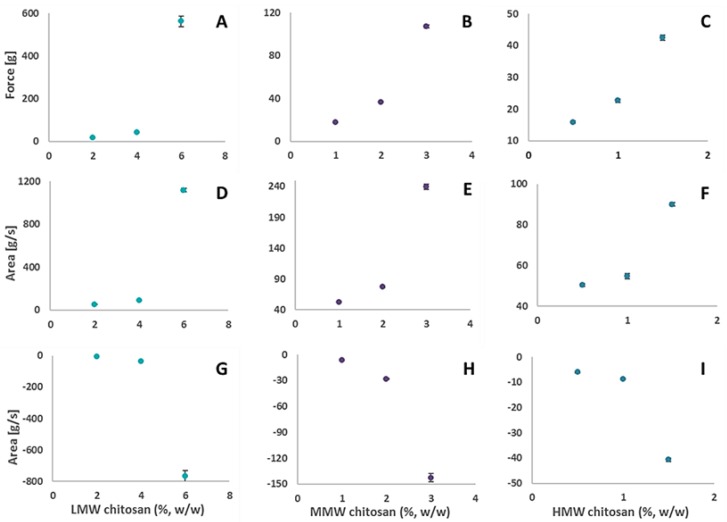
Texture analysis determining the hydrogel hardness (**A**–**C**), cohesiveness (**D**–**F**) and adhesiveness (**G**–**I**) for low molecular weight (LMW), medium molecular weight (MMW) and high molecular weight (HMW) chitosan hydrogels (%, *w*/*w*).

The polymer molecular weight will affect the properties of the chitosan hydrogel [40]. High, medium, and low molecular weight chitosan were used to prepare hydrogels with various polymer concentrations and the hydrogel hardness, cohesiveness, and adhesiveness measured to optimize the molecular weight and polymer concentration (Figure 2). High molecular weight (HMW) polymers have several cross-linkages per polymer, resulting in a more robust and stiff hydrogel [40]. Even at lowest chitosan concentrations, the HMW hydrogel was found too rigid for vaginal application and the texture properties were poor at the lower polymer concentrations. The lower molecular weight chitosan has expressed superior mucoadhesive properties compared to HMW [41]; however, a rather high polymer concentration was needed to assure desired mechanical properties. On the other hand, the hydrogels made of MMW chitosan were found to be most suitable for vaginal administration and their texture properties were found adequate for vaginal administration, thus, MMW hydrogels were used in further experiments. 

**Figure 3:**
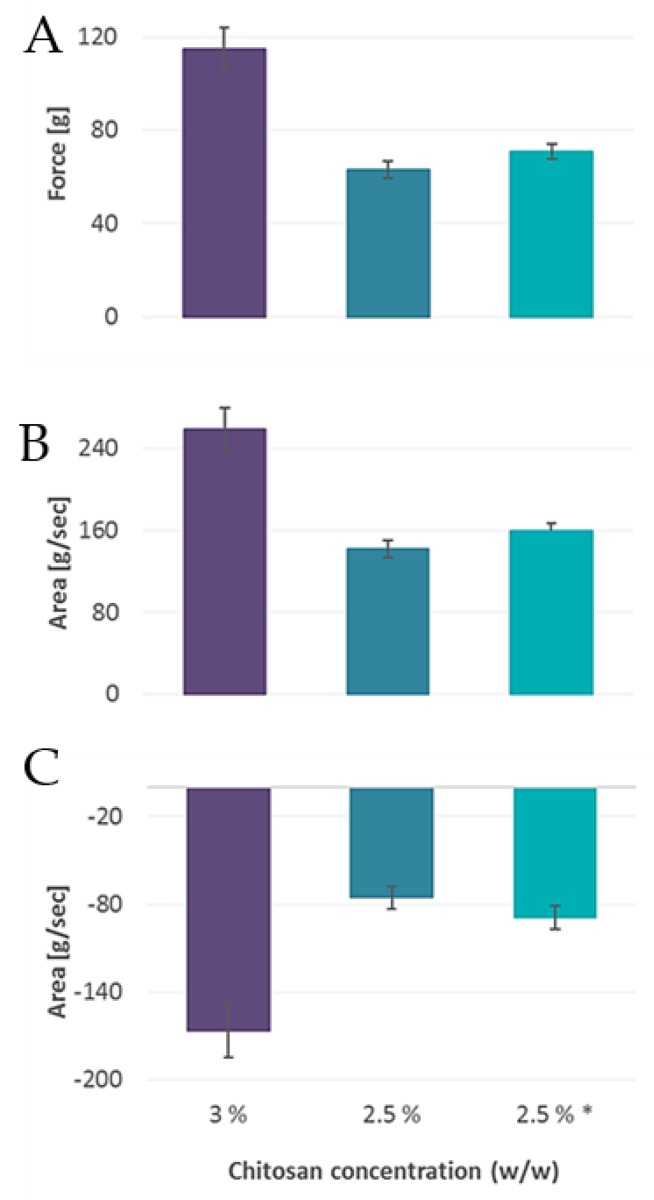
Texture analysis determining the hydrogel hardness (**A**), cohesiveness (**B**), and adhesiveness (**C**) for chitosan hydrogels (2.5 and 3%, *w*/*w*, *n* = 3). * Texture stability of hydrogel after two months storage at 4–8 °C.

Chitosan concentrations of 3 and 2.5% (*w*/*w*), respectively, were further investigated. Texture analysis is an important tool in the formulation development, including the stability studies, enabling the determination of gel texture under the same experimental setup. The indications of changes in gel hardness, cohesiveness and adhesiveness correlate well with the loss of gel’s stability. The stability of the hydrogel expressed as the changes in hardness, cohesiveness and adhesiveness was measured, and confirmed that textural properties were maintained during the storage (4–8 °C) for a period of two months (Figure 3).

**Figure 4:**
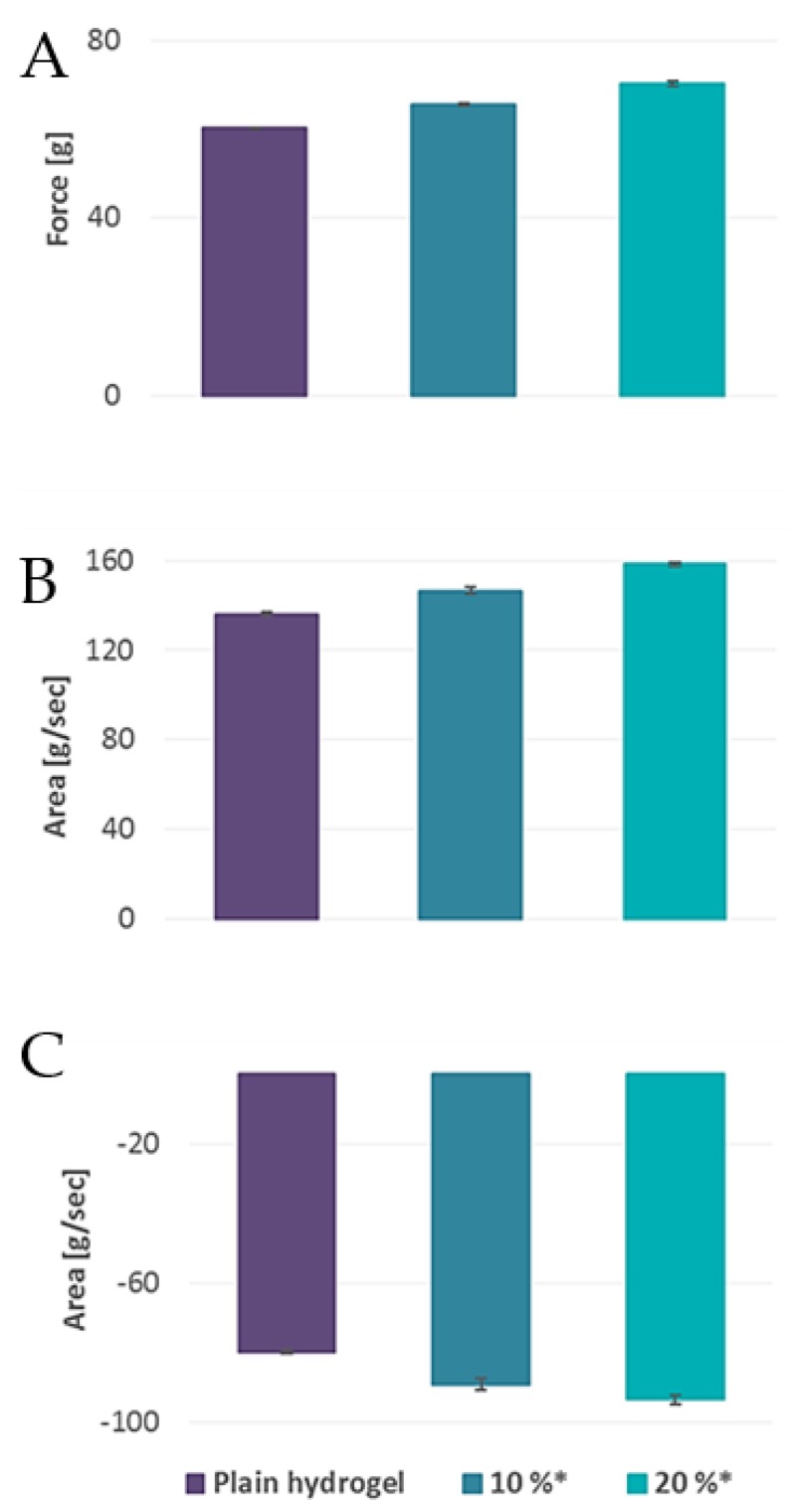
Texture analysis determining the hydrogel hardness (**A**), cohesiveness (**B**), and adhesiveness (**C**) for chitosan hydrogels (2.5% *w*/*w*) with and without liposomes (*n* = 3). * Concentration of liposomal suspension incorporated into hydrogel (%, *w*/*w*).

Incorporation of liposomes in hydrogels can affect the rheology of hydrogels [13]. Hydrogels incorporating liposomes (10% and 20%, *w*/*w*, respectively) were prepared to follow the effect of liposomes on the hydrogel texture properties. Rather interestingly, all parameters (hydrogel hardness, cohesiveness and adhesiveness), were increased for hydrogels containing liposomes (Figure 4). The increased mechanical properties were proportional with the increase in concentration of incorporated liposomes. In a comparison, we measured the control hydrogels containing propylene glycol (RES) and buffer (EPI) in concentration corresponding to liposomal suspensions, all with same polymer concentration (2.5%, *w*/*w*). Chitosan hydrogels containing buffer (liposome free, 20% buffer) showed a clear reduction in texture properties (data not included in figure: hardness: 59.79 g; cohesiveness: 134.28 g/s; adhesiveness: –69.52 g/s). However, the incorporation of propylene glycol (10%) instead of liposomal suspensions resulted in hydrogels exhibiting superior properties, both compared to plain hydrogel and hydrogel containing liposomes (data not included in figure: hardness: 82.06 g; cohesiveness: 185.92 g/s; adhesiveness: –111.96 g/s). The results are clear indication that original hydrogel texture properties are affected by the incorporation of liposomes or solvents in their network. Liposomal suspensions assured gel stability, whereas propylene glycol had a positive effect on the gel network.

It was earlier reported that the texture properties of liposomes-in-hydrogel formulations affected by both liposomal size and their surface charge [33]. We did not elaborate on these parameters further, as our liposomes were of similar size and neutral.

A gel prepared with chitosan concentration of 3% (*w*/*w)* expressed superior texture properties compared to hydrogels containing lower chitosan concentration (2.5%, *w*/*w*, Figure 3). However, in respect to comfort and ease of application, lower chitosan concentration appeared more appropriate. Thus, the mucoadhesiveness was evaluated for both formulations. Although the texture analyzer allows the use of parameters fitting the physiological conditions, the variations in the force applied, contact time and withdrawal speed makes it difficult to compare the mucoadhesiveness for formulations and mucoadhesive polymers [42]. Hence, the ex vivo mucoadhesiveness of the liposome-in-hydrogel formulations was assessed by two separate experiments; The detachment force was measured, and to provide additional insight on the formulations mucoadhesiveness, the amount of formulation left on the tissue was also determined [22]. Further, the same parameters were applied in all measurements. The results confirmed similar mucoadhesive properties for both hydrogels (2.5 and 3%, *w*/*w*) and imply that lower chitosan concentration does not compromise the mucoadhesiveness of the formulation (Table 2). Hence, the chitosan concentration of 2.5% (*w*/*w*) was used for further experiments.

#### 3.2.2. Polyphenol Release Studies

**Figure 5:**
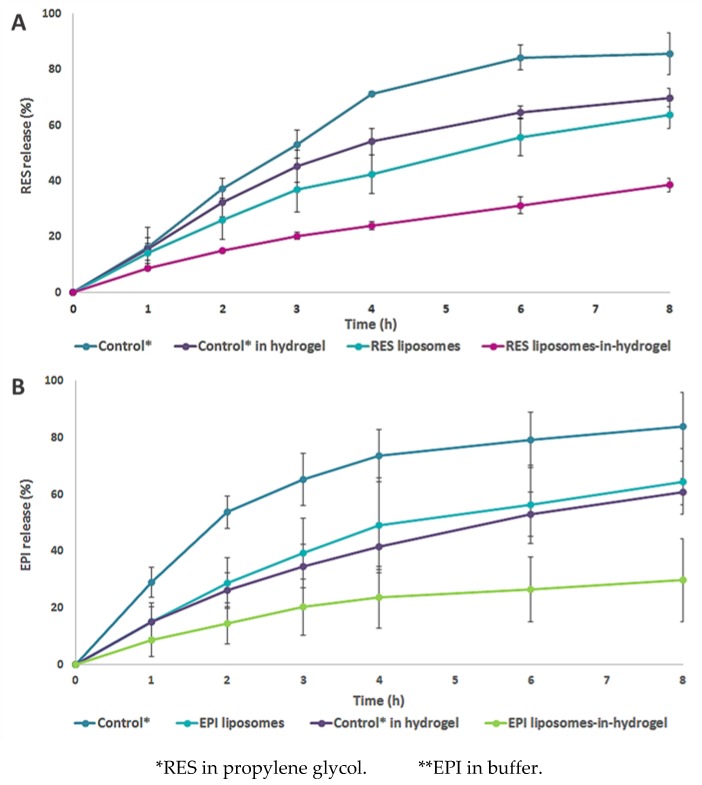
RES (**A**) or EPI (**B**) release from liposomes and liposomes-in-hydrogel compared to the respective controls. Results are expressed as percentage mean ± SD (*n* = 3).

The in vitro release of polyphenols was measured on a Franz cell diffusion system, which is considered one of the most suitable methods for evaluating the release from semisolid topical dosage forms, including gels intended for vaginal application [43,44]. The studies were performed in the mimicked vaginal environment with a receptor medium mimicking the pH of healthy human vaginal condition (4.6) and a temperature of 37 °C. The in vitro RES release from the liposome-in-hydrogel formulation was compared to the release of free RES (RES in propylene glycol), liposomal RES and free RES in hydrogel. A sustained release was determined from both liposomal and liposomes-in-hydrogel formulations compared to the respective controls (Figure 5A). RES liposomes sustained the RES release significantly already after four hours (*p* < 0.03). By incorporating RES solution (propylene glycol) into the hydrogel, a prolonged release was also attained. However, the combination of the two delivery systems expressed a more pronounced sustained release. The release from RES liposomes-in-hydrogel was also shown to be consistent, with very small variations between the experiments, and was significantly sustained already after two hours (*p* < 0.01). A similar release profile was determined for EPI, with the prolonged release from hydrogel formulations compared to the respective controls, and the enhanced sustained effect when liposomes and hydrogel were combined within one delivery system (*p* < 0.03; Figure 5B). Considering the potential biological activities of RES and EPI, respectively, the concentrations of released polyphenols are above the effective concentrations required to induce desired biological effects.

In the evaluation of *ex vivo* polyphenol penetration, vaginal tissue from pregnant sheep was used [20]. The experiments were performed in the presence of vaginal fluid simulant (VFS) to further mimic the in vivo vaginal condition.

**Figure 6:**
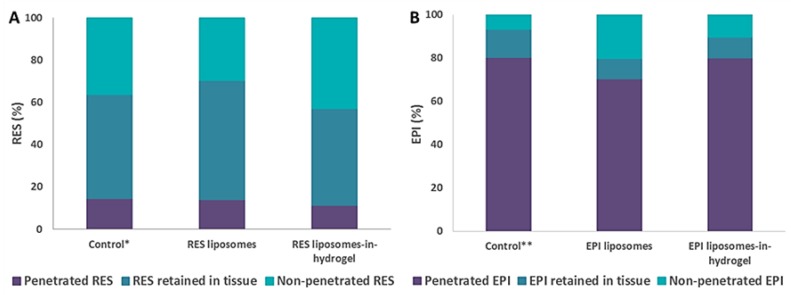
RES (**A**) and EPI (**B**) partitioning from liposomes and liposomes-in-hydrogel, after 8 h of ex vivo penetration experiment as compared to the respective controls. Results are expressed as percentage mean (*n* = 3). * RES in propylene glycol. ** EPI in buffer.

The ex vivo polyphenol penetration results indicate a minor RES penetration of 14.46 ± 1.59 for control, 14.05 ± 2.92 for RES liposomes and 11.42 ± 5.56 for RES liposomes-in-hydrogel, respectively. The differences were not significant (Figure 6A). Majority of RES appear to retain within or on top of tissue after eight hours of experiment. Even though propylene glycol is known to enhance skin penetration, free RES in propylene glycol did not penetrate the tissue to a greater extent, in agreement with penetration seen for other compounds [20]. This indicates that topical administration of RES will avoid considerable systemic absorption, probably due to a pronounced binding to the mucosal tissue and mucus [4]. However, a pronounced EPI penetration of 80.30 ± 7.42 for control, 70.45 ± 11.67 for EPI liposomes, and 79.10 ± 4.41 for EPI liposomes-in-hydrogel was detected respectively (Figure 6B). Only minor amounts of EPI retained within and in the vaginal tissue. We did not have an opportunity to further study potential penetration of EPI via vaginal tissue; to provide a deeper insight on the penetration pathways, if indeed existing, would require animal experiments. However, the observed finding should be further examined.

### 3.3. Effect of Polyphenol Liposomes-In-Hydrogel on Cell Toxicity

**Figure 7:**
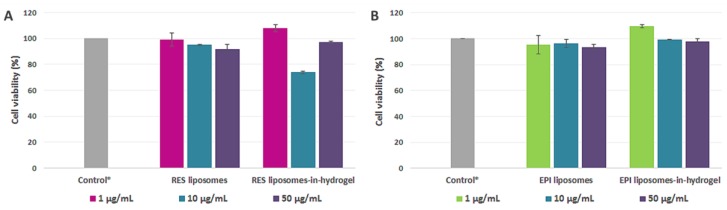
Cytotoxic effects of RES- (**A**) and EPI- (**B**) liposomes or liposomes-in-hydrogel on HaCat cell viability compared to the viability of untreated cells (100%). Results are expressed as percentage mean ± SD (*n* = 3).

The effects of polyphenol formulations on cell toxicity were evaluated and results are presented in Figure 7. The cytotoxicity results are expressed as percentages of viable cells when the cells were exposed to the formulations for 24 h, compared to the viability of the untreated cells (100% media only). Therefore, higher percentage of cell viability means lesser toxic effects of the RES or EPI formulations. RES liposomes exhibited a negligible influence on the cell toxicity compared to the untreated cells. For the RES liposomes-in-hydrogel, a reduced cell viability was detected at the concentration of 10 µg/mL, however, both at higher (50 µg/mL) and lower (1 µg/mL) concentrations no cytotoxic effects were observed. 

Both EPI liposomes and EPI liposomes-in-hydrogel expressed no effect on cell toxicity up to the concentration of 50 µg/mL (Figure 7). All tested concentrations showed similar cell viability as non-treated cells (control). These preliminary cytotoxic results suggest that both RES and EPI liposomes-in-hydrogel are not toxic to living cells at the concentration up to 50 µg/mL.

### 3.4. Anti-Oxidative Activity of EPI 

**Figure 8:**
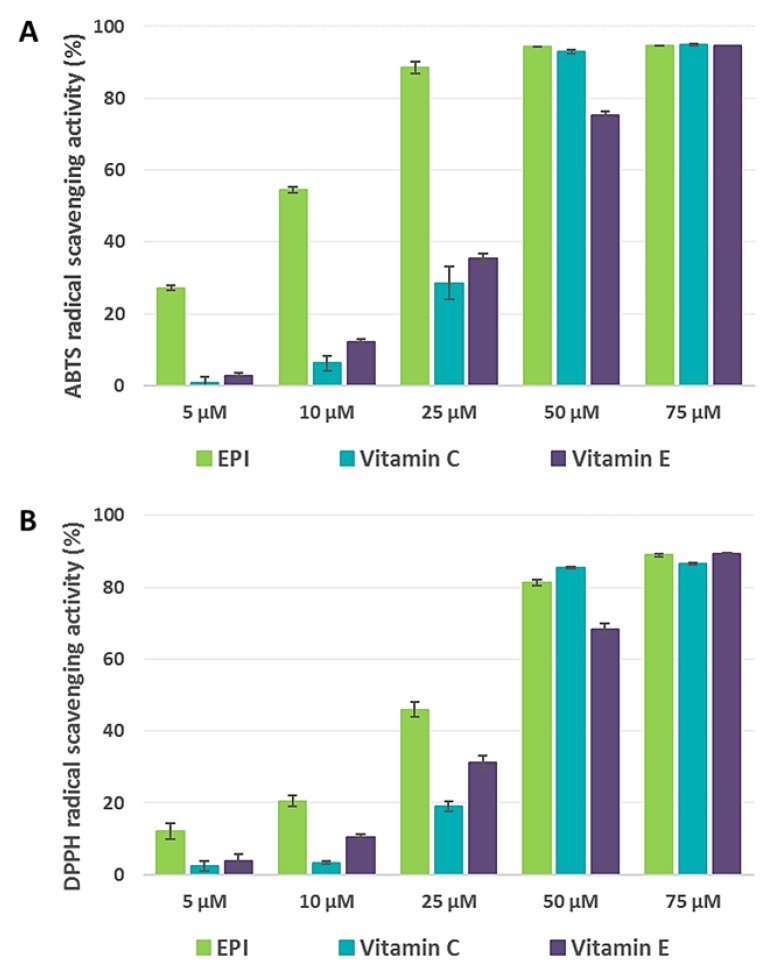
Anti-oxidative activities of EPI expressed as ABTS (**A**) and DPPH (**B**) free radical scavenging activity comparing to vitamins C and E. Results are expressed as mean ± SD, (*n* = 3).

Polyphenols are known to exhibit anti-oxidative and anti-inflammatory activities [45]. We have previously reported the anti-oxidative activity of RES [5], confirming its potent ABTS radical scavenging activity in comparison to vitamin C and E. The results of anti-oxidative activities of EPI are shown in Figure 8 in terms of ABTS and DPPH free radicals scavenging activities. Comparing to the well-known antioxidants such as vitamins C and E, EPI showed significantly stronger activity against both ABTS and DPPH free radicals at the lower concentrations (Figure 8). All tested substances showed a concentration dependent scavenging activity; however, EPI showed six- to 10-fold stronger radical scavenging activity comparing to vitamin C and E at lower concentrations.

Our results also support earlier reports that EPI has strong potential to eliminate the toxic effect of free radicals generated in the biological systems especially during the inflammation and infection [46,47]. Moreover, deeper understanding of the activity of liposomal EPI on free radicals could be strengthen by applying fluorescents probes as suggested by Lucio and colleagues [48]. Radical scavenging activities evaluations performed in this work are simple chemical tests, therefore, pure chemical components instead of corresponding formulations were tested.

### 3.5. Anti-Inflammatory Activity of EPI

Anti-inflammatory activities of both RES and EPI are sufficiently reported, however not when polyphenols are incorporated in formulations such as hydrogels. Current polyphenol-based liposome-in-hydrogel formulations are aimed for the treatment of vaginal inflammation and infection. There are sufficient scientific evidences that NO has a central role during inflammation and infection. The LPS-induced macrophages can produce a toxic amount of NO causing inflammation. Control of NO overproduction can be beneficial to reduce excessive inflammation. Therefore, collecting evidences on the targeted drugs/active substances and corresponding formulations regarding their NO modulating activities is an important step. We have already reported the effects of RES and liposomal RES on inhibition of NO production in LPS-induced macrophages [5], and we found that RES expressed a concentration dependent NO inhibitory activity. Furthermore, liposomal RES was found to be more potent than RES in solution [5].

**Figure 9:**
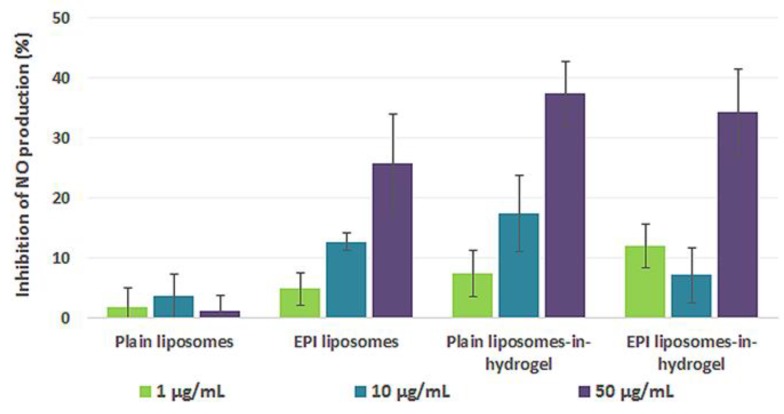
Inhibitory effect of EPI liposomes-in-hydrogel on NO production compared to plain liposomes-in-hydrogel. Results are expressed as percentage mean ± SD (*n* = 3).

In this paper, we are reporting the anti-inflammatory effects of EPI formulations by measuring the inhibitory effects on NO production. The amount of NO produced by the macrophages by inducing LPS (1 µg/mL) is considered as the control. Compared to the control group, the reduction in NO production by liposomes alone and polyphenol-containing formulations as treatment groups, under similar conditions, were expressed as percentage inhibition. Comparing to plain liposomes, EPI liposomes showed significantly higher percentage of inhibitions, demonstrating clearly that EPI contributes to inhibition of NO production. The results presenting the inhibitory effects of plain liposomes-in-hydrogel and EPI liposomes-in-hydrogels on NO production in LPS-induced macrophages are shown in Figure 9. Both EPI liposomes-in-hydrogel and plain liposomes-in-hydrogel exhibited a strong inhibitory effects on NO production in a concentration-dependent manner comparing to control and they were not significantly different. The results indicated that anti-inflammatory activities are contributed to the chitosan hydrogel alone. The reason that EPI containing hydrogel did not exhibit further inhibition comparing to hydrogel alone might be due to already attained maximum inhibition; the finding requires further study. 

Hydrogels as vehicles destined for vaginal administration are considered superior delivery systems [49]. It was recently reported that chitosan increases the penetration and adhesion of imiquimod through vaginal mucosa [50]. This would be additional argument to use chitosan as a mucoadhesive vehicle for localized vaginal administration. Earlier studies have also shown that chitosan inhibits LPS-induced inflammatory effects in RAW 264.7 macrophages [51]. Chitosan is not only assuring longer retention time at the vaginal site, it also contributes to anti-inflammatory and antimicrobial potential of liposomes-in-hydrogel formulations.

## 4. Conclusions

Herewith we developed a novel liposomes-in-hydrogel formulation for polyphenols of different structure having similar biological activities. The incorporation of liposomes into chitosan hydrogel improved gel’s texture properties. The liposome-in-hydrogel delivery system expressed good mucoadhesive properties, indicating that a prolonged retention time at administration site is achievable. The novel system provided a controlled and prolonged release of entrapped polyphenols. Current formulations did not show any direct toxic effect to live cells at considerable treatment concentration. This is an indication that the system is non-toxic, however, further in vivo studies are needed to confirm its safety. EPI showed potent anti-oxidative activity and an enhanced anti-inflammatory activity at higher concentration when formulated in liposomes-in-hydrogel system. Chitosan contributes to the system’s biological activities; therefore, chitosan-based hydrogels have clear potential as a vaginal delivery system for localized infections and inflammation.

## Figures and Tables

**Table 1:** Liposomal size, size distribution, zeta potential, and entrapment efficiency of RES- or EPI- containing liposomes.

	Vesicle Size (nm)	PI *	Zeta Potential (mV)	Entrapment (%)	Polyphenol/Lipid Ratio (µg/mg)
RES liposomes	192 ± 15	0.100	−3.42 ± 1.02	81 ± 10	54.29 ± 2.33
EPI liposomes	196 ± 13	0.072	−3.32 ± 1.06	77 ± 2	48.48 ± 3.05

Results are expressed as mean ± SD (*n* = 3). * Polydispersity index.

**Table 2:** Detachment force and formulation retained on the tissue for chitosan hydrogels (2.5 and 3%, *w*/*w*).

	RES Liposomes		EPI Liposomes	
Chitosan concentration (%, *w/w*)	2.5	3	2.5	3
Detachment force [g]	10.66 ± 1.59	11.01 ± 1.43	9.82 ± 1.39	11.88 ± 0.43
Formulation retained on tissue (%)	73 ± 5	72 ± 3	79 ± 5	70 ± 3

Results are expressed as mean ± SD (*n* = 3).

## Abbreviations

ABTS	2′-azino bis(3-ethylbenzothiazoline)-6-sulfonic acid diammonium salt
DPPH	1-diphenyl-2-picrylhydrazyl
EPI	epicatechin
LPS	lipopolysaccharide
NO	nitric oxide
PBS	phosphate buffer saline
PC	phosphatidylcholine
PI	polydispersity index
RES	resveratrol
STI	sexually transmitted infections
